# Blackcurrant Leaf Chlorosis Associated Virus: Evidence of the Presence of Circular RNA during Infections

**DOI:** 10.3390/v10050260

**Published:** 2018-05-15

**Authors:** Delano James, James Phelan, Daniel Sanderson

**Affiliations:** Sidney Laboratory, Centre for Plant Health, Canadian Food Inspection Agency, 8801 East Saanich Road, North Saanich, BC V8L 1H3, Canada; james.phelan@inspection.gc.ca (J.P.); daniel.sanderson@inspection.gc.ca (D.S.)

**Keywords:** *Idaeovirus*, blackcurrant leaf chlorosis associated virus, next-generation sequencing (NGS), bridge reads, abutting primers, RNase R digestion, circular RNA, concatenated RNA

## Abstract

Blackcurrant leaf chlorosis associated virus (BCLCaV) was detected recently by next-generation sequencing (NGS) and a new and distinct species in the genus *Idaeovirus* was proposed. Analysis of NGS-derived paired-end reads revealed the existence of bridge reads encompassing the 3′-terminus and 5′-terminus of RNA-2 or RNA-3 of BCLCaV. The full RNA-2 or RNA-3 could be amplified using outward facing or abutting primers; also, RNA-2/RNA-3 could be detected even after three consecutive RNase R enzyme treatments, with denaturation at 95 °C preceding each digestion. Evidence was obtained indicating that there are circular forms of BCLCaV RNA-2 and RNA-3.

## 1. Introduction

Blackcurrant leaf chlorosis associated virus (BCLCaV) was detected by next-generation sequencing (NGS) and analysis of its genome indicated that it is a new member of the genus *Idaeovirus* [1]. A similar virus described as blackcurrant idaeovirus (BCIV), likely an isolate of BCLCaV, was detected and characterized also by Thekke-Veetil et al. [2]. Several genomic components associated with BCLCaV were identified by NGS and/or RT-PCR and confirmed by RT-PCR where necessary [1]. BCLCaV genomic components include: (1) RNA-1 that is monocistronic with a single open reading frame (ORF1) that encodes the replicase complex (M_r_ 197 kDa); (2) a bicistronic RNA-2 that encodes a putative movement protein (MP, ORF2a, M_r_ 38.8 kDa) and the putative coat protein (CP, ORF2b, M_r_ 30 kDa) of the virus, with ORFs that overlap by a single adenine (A) nucleotide (nt), representing the third position of an opal stop codon of ORF2a and the first position of the start codon of ORF2b; (3) a subgenomic form of RNA-2 (putative RNA-3) that contains ORF2b, the putative CP coding region; and (4) a concatenated form of RNA-2 that consists of an inverted RNA-3 conjoined to the full-length RNA-2 [1,2]. Navarro et al. [3] recently described a novel virus in privet, privet leaf blotch-associated virus (PrLBaV) and proposed that PrLBaV be recognized also as a species in the genus *Idaeovirus*.

*Raspberry bushy dwarf virus* (RBDV) is a linear, positive sense ssRNA virus, the only species of the genus *Idaeovirus* recognized currently by the International Committee on Taxonomy of Viruses (ICTV) and the type member of the genus [4]. RBDV was first described by Cadman in 1961 [5]. The genome sequence of the virus (RNA-1, RNA-2, RNA-3) was described approximately 30 years later [6,7,8]. In 1993, it was proposed that RBDV be classified in the genus *Idaeovirus* [9] and since then, for over 20 years, has been the only ICTV-recognized species in the genus. RBDV RNA-1 ORF1 encodes a 190 kDa protein [8]; the RNA-2 is bicistronic with ORF2a encoding a 39 kDa protein (with similarity to cell-to-cell virus movement proteins) and ORF2b encoding a 30 kDa protein, the CP of the virus [4,7,9]; and a subgenomic RNA-2-derived fragment that is likely involved in CP expression and designated RNA-3 [4,7,9]. In agroinfiltration studies of RBDV, MacFarlane and McGavin [10] showed that inclusion of the subgenomic RNA-3 fragment, along with transcripts of the RNA-1 and RNA-2, contributed to genome activation, resulting in greatly increased levels of infection.

RBDV, PrLBaV, and BCLCaV are closely related phylogenetically; they possess similar genome organizations expressing similar proteins of similar sizes and their 3′ terminal nucleotide (nt) sequences form typical stem loops and are terminated by a 3′ poly(C) tail [1,2,3,7], supporting classification of the latter two viruses as members of the genus *Idaeovirus*. There are certain peculiarities associated with these viruses. Their RNA-2 intergenic noncoding regions (IG-NCR) vary in size, with approximately 117 nt for RBDV (accession NC_003740), 1 nt for PrLBaV (LT221869), and 0 nt for BCLCaV (KX838924). The third nt (A) of the opal stop codon (UGA) of BCLCaV ORF2a acts as the first nt (A) of the start codon (AUG) of ORF2b [1,2], a phenomenon described as “termination–reinitiation” [11]. Quito-Avila et al. [12] described the detection of a modified RNA-2 fragment (RNA-2m) associated with an Ecuadorean isolate of RBDV found in blackberry (*Rubus glaucus*). James and Phelan [1] described also detection of a BCLCaV RNA-2 concatenated genomic component similar to the RNA-2m described by Quito-Avila et al. [12], but without any IG-NCR between ORF2a and ORFb2 in the case of BCLCaV. Due to its inverted and complementary ends, RNA-2m is presumably able to form a panhandle-type loop [12].

Herold and Andino [13] suggested that RNA circularization may be a general replication mechanism used by positive sense RNA viruses. Until recently, circular RNAs were considered to be rare anomalies but NGS analyses—also known as high-throughput or deep sequencing—has resulted in the discovery of an abundance of stable circular RNAs that are often the products of RNA splicing and may be involved in gene expression [14,15,16,17]. Circular RNAs are difficult to detect and may have eluded identification in the past [18]. They may consist of noncoding or coding RNA and can be quite variable in size, 100s to 1000s of nucleotides in length [16,17,19,20]. Viroids are circular noncoding RNA that can be pathogenic in plants [19,21]. It appears that there are several mechanisms by which RNAs may become covalently linked or circularized, including back-splicing, direct 3′–5′ ligation, and mediation by ribozymes [22,23].

In this study, BCLCaV RNA-2/RNA-3-derived “bridge reads” were observed among NGS-generated paired-end reads generated from dsRNA extracted from BCLCaV-infected blackcurrant (*Ribes nigrum*). Bridge reads suggestive of circularity were found in essentially two types. One type included the 3′ terminus and 5′ terminus of BCLCaV RNA-2 as contiguous sequences and the other featured the 3′ terminus and 5′ terminus of BCLCaV RNA-3. We hypothesized that this may indicate the presence of other forms of end-to-end concatenated RNA-2/RNA-3 associated with BCLCaV, or possibly circular RNA forms. RT-PCR assays were developed to confirm linkages of the 3′ terminus and 5′ termini, as indicated by the NGS-derived bridge reads. Techniques were used to determine if indeed circular RNA was present including: (a) attempts at amplification of full-length fragments using outward facing or abutting primers [24,25] and (b) RNase R treatments [18,26,27] followed by RT-PCR. Highly specific and efficient amplification techniques, such as touchdown RT-PCR [28,29], were combined with reagents such as SuperScript III reverse transcriptase and Platinum Hi-Fi *Taq* DNA polymerase to aid in identifying concatenation. The results of these studies are described. Evidence is presented that indicates that circular RNAs are associated with BCLCaV RNA-2 and RNA-3.

## 2. Materials and Methods

### 2.1. Virus Source

Blackcurrant leaf chlorosis associated virus (BCLCaV) was maintained in blackcurrant plants (*Ribes nigrum* cv. Baldwin), accession number 3124-03D1, co-infected with the potexvirus Actinidia virus X [30,31]. BCLCaV isolate 3124-03D1 was transferred to *Nicotiana benthamiana* by mechanical sap-transmission, as described by James and Phelan [30]. Healthy *Ribes* plants and healthy *N. benthamiana* plants were used as negative controls.

### 2.2. Double-Stranded RNA Extraction, Complementary (c)DNA Library Creation, and Illumina NGS Sequencing

Double-stranded RNA (dsRNA) extractions from symptomatic *R. nigrum* cv. Baldwin (accession 3124-03D1) and from a healthy control plant were carried out as described by Tzanetakis and Martin [32]. Complementary DNA (cDNA) libraries were prepared and NGS sequencing was carried out as described by James and Phelan [1].

### 2.3. Total RNA Extractions

Total RNA extractions from *N. benthamiana* and blackcurrant were carried out using QIAGEN’s RNeasy Plant Mini Kit (cat. # 74904, QIAGEN, Toronto, ON, Canada) using the modified protocol described by Kalinowska et al. [33]. Total RNA was eluted in 50 µL diethylpyrocarbonate-treated H_2_O.

### 2.4. Complementary (c)DNA Production, Cloning, Sanger Sequencing

Primer design was aided by Clone Manager 9 (Professional Edition (c) 1994-2010, Scientific and Educational Software, Cary, NC, USA). cDNA was generated from total RNA using Invitrogen’s SuperScript II^TM^ reverse transcriptase and the appropriate oligonucleotide primers (denaturation phase 5 min at 94 °C; synthesis phase 1 h at 42 °C). This was followed by PCR performed on the cDNA template using Invitrogen Platinum^®^ *Taq* DNA Polymerase High Fidelity. Amplified cDNA fragments were gel purified and extracted using a MinElute Gel Extraction Kit (QIAGEN), ligated into the pCR 2.1 TOPO vector, and cloned using the TOPO TA Cloning Kit as described by the supplier (Invitrogen, Carlsbad, CA, USA). Plasmids with virus-derived cDNA inserts were sequenced by Sanger sequencing at the Nucleic Acid Protein Service Unit, UBC, (Vancouver, BC, Canada), on an Applied Biosystems 3730 DNA Analyzer (Thermo-Fisher, Waltham, MA, USA).

### 2.5. Genome Assembly and Sequence Analysis

Raw data files were imported, paired, and the reads trimmed with default parameters for quality and ambiguous nucleotides. Additionally, reads were trimmed of TruSeq universal and indexed adapter sequence. Bridge reads were identified by read mapping to BCLCaV sequences with increasing stringency using CLC Genomics Workbench v7.5.1 (CLCBio, www.clcbio.com). Alignments were generated in CLUSTAL-X 2.1 [34,35]. Folding analysis of genome fragments was carried out using both the mfold Web Server at http://mfold.rit.albany.edu [36] and RNAfold, Vienna Web Server [37].

### 2.6. BCLCaV Diagnostic RT-PCR

**Figure 1:**
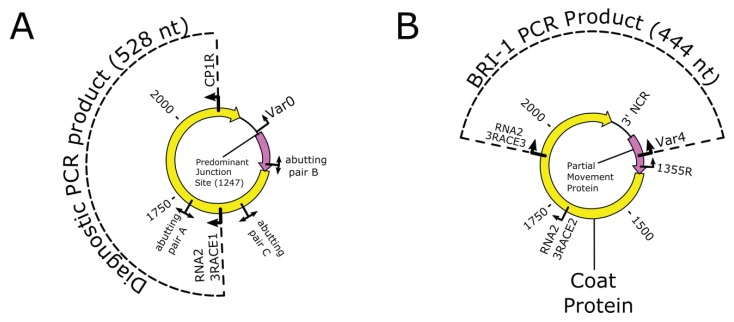
Diagrams representing a circular blackcurrant leaf chlorosis virus (BCLCaV) RNA-3 showing: (**A**) the position of the BCLCaV diagnostic RT-PCR primers for the 528 nt product and position of abutting primer pairs and (**B**) the position of bridging RT-PCR primers spanning the 3′ terminus and 5′ terminus of the putative circular RNA-3 to confirm bridging by nested RT-PCR.

A two-step BCLCaV-specific RT-PCR was developed for diagnosis to allow reliable detection of the virus [1]. The RT-PCR utilized the forward primer RNA2_3RACE1 and the reverse primer CP1R (Table 1, Figure 1A). These primers target ORF2b that encode the coat protein (CP) coding region of RNA-2 (present also in RNA-3) of BCLCaV and amplify a 528 bp fragment. Amplification reactions were carried out using an Eppendorf^®^ Mastercycler^®^ Pro S machine with conditions as described by James and Phelan [1]. When necessary for confirmation, amplified cDNA fragments were gel purified, cloned, and at least three independently derived clones were Sanger sequenced as described above.

### 2.7. RT-PCR to Verify NGS Generated Bridge Reads

In order to verify the “bridge reads”—identified in the NGS data—that connected the 3′ terminus of RNA-2/RNA-3 to the 5′ terminus of the respective RNAs, RT-PCR tests were designed using outward facing primers (with respect to the linear RNA) that would flank a hypothesized junction site. The samples used in these tests included total RNA samples extracted from BCLCaV-infected *Ribes nigrum* and *Nicotiana benthamiana* tissue, as well as healthy controls, as described above. Multiple strategies were used, including: (a) RT-PCR with primer pair BRI-0 (Table 2, Figure 1B) used to amplify a fragment spanning the 3′ and 5′ termini of RNA-2, if linkage exists (Figure 1B shows RNA-3, primer RNA2 5RACE3 is in the same orientation as Var4 but upstream, so is not shown); (b) nested RT-PCR using the external primers BRI-4 for the first amplification, followed by a second round of amplification using the internal primer pair BRI-1; (c) RT-PCR using primer pair BRI-4; and (d) RT-PCR using primer pair BRI-1 (Table 2, Figure 1B). The RT-PCR tests were performed as two-step reactions, with the cDNA synthesis steps using SuperScript III reverse transcriptase kits, and PCR steps using Platinum Hi-Fi *Taq* DNA polymerase (Thermo-Fisher, Waltham, MA, USA). For the cDNA synthesis reaction, a 10 μL pre-mix containing 2.5 nmol of each dNTP, 0.01 nmol of a gene-specific primer, and 1 μL of total RNA template was denatured at 65 °C for 5 min, then placed on ice immediately. The pre-mixes were then added to 10 μL of a cDNA synthesis mix (which contained reaction buffer, DTT, MgCl_2_, Superscript III, and RNase OUT at the manufacturer’s recommended levels) to give a final 20 μL reaction mix. Reverse transcription was carried out at 50 °C for 50 min. When completed, 2 μL of this product was used for PCR amplification. These reactions (25 μL) used gene-specific primers at a final concentration of 0.4 μM and MgSO_4_ at 1.5 mM, with other reagents used as recommended by the manufacturer. PCR was preceded by a 2 min denaturation at 94 °C, followed by a touchdown phase of 17 cycles. In this phase, each denaturation was at 94 °C for 30 s, followed by a 30 s annealing step for which the temperature was set at 1 °C lower than that of the previous cycle, starting from 72 °C. Elongation steps were at 68 °C for 1 min/kb of target. For the PCR phase, cycles were identical to that of the touchdown phase, except for the annealing temperature, which was kept at 55 °C for over 25 cycles. In cases where a second round of PCR was performed, 2 μL of the previous PCR reaction was used in a similar 25-cycle reaction with no touchdown phase, and with final primer and MgSO_4_ concentrations at 0.2 μM and 2 mM, respectively.

### 2.8. RT-PCR with Abutting Primers

An effective strategy used commonly for detecting the presence of circular nucleic acid forms is amplification using abutting primers to produce full-length fragments [24,39]. The sequences of the abutting primers that target the BCLCaV CP coding region of RNA-2/RNA-3 are given in Table 3, and their positions are shown in Figure 1A. These primers will detect RNA-2 and/or RNA-3 because of their location. Tests were done on both total RNA and dsRNA extracts of BCLCaV-infected plant tissue, in a manner similar to that for the RT-PCR tests mentioned previously. In these tests, 5 μL of template was used in the cDNA pre-mix; dNTPs in this case were added to the cDNA synthesis mix, and Superscript II was used as the reverse transcriptase (Thermo-Fisher, Waltham, MA, USA). The PCR reaction was performed over 35 cycles with cycling conditions described in the previous section but omitting the touchdown phase. Standard *Taq* polymerase and *Taq* Extender reagents were used here at the manufacturer’s recommended concentrations (Thermo-Fisher, Waltham, MA, USA). Final concentrations of other reagents were: 1.5 mM MgCl_2_, 0.1 mM dNTP, and 0.2 μM each of the forward and reverse primers.

### 2.9. RNase R Enzyme Treatments Followed by RT-PCR Analysis

**Figure 2:**
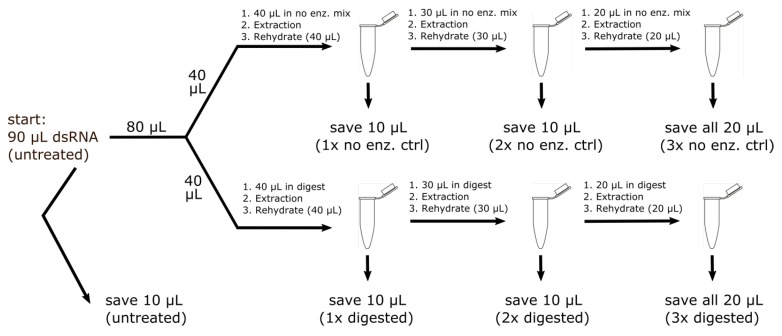
RNase R treatments of dsRNA samples, where three consecutive digests were carried out, with 10 μL samples saved at each step for RT-PCR screening (**lower branch**), and a parallel no-enzyme control series (**upper branch**) to validate the procedure. Denaturation at 95 °C for 8 min was carried out before each RNase R treatment.

RNase R digestion was performed using a procedure similar to that described by Suzuki et al. [27], but with digestion at 37 °C for 45 min. The workflow diagram (Figure 2) summarizes the RNase R assays conducted, indicating for each sample three rounds of denaturation followed by RNase digestion after each step. Treatments were replicated 2–4 times. Double-stranded RNA (dsRNA) was isolated as described above. Samples were adjusted to a total starting volume of 90 μL, of which 10 μL was saved as an untreated control. The remaining 80 μL volume was split evenly between: (1) a set of consecutive RNase R treatments (Figure 2, lower branch) and (2) a set of control treatments handled in the same fashion but with the RNase R replaced by an equivalent volume of water, referred to as the no-enzyme controls (Figure 2, upper branch). Before each treatment, a denaturation step (95 °C for 8 min) was carried out before addition of the RNase R enzyme (or water, in the case of the no-enzyme control). RNase treatments and the no-enzyme treatments were carried out in 50 μL volume reactions at 37 °C for 45 min. After each treatment, a phenol chloroform extraction of the nucleic acid was performed, and the volume readjusted with water to a volume similar to that of the original reaction. Analysis using the diagnostic RT-PCR described above was performed on each set of the retained nucleic acid samples (Figure 2). RT-PCR targets included BCLCaV RNA-1, BCLCaV RNA-2/RNA-3, a diagnostic RT-PCR for the co-infecting virus Actinidia virus X (AVX; [31]), and an RT-PCR for the mRNA of the host mitochondrial *nad*5 gene [38]. The sequences of the primers used in the post-digestion RT-PCR reactions are listed in Table 1. RT-PCR products that were needed for confirmation were excised, gel extracted using a QIAGEN MinElute kit, cloned as described earlier using the TOPO-TA cloning technique (Thermo-Fisher) and Sanger sequenced.

## 3. Results

### 3.1. Sequence Data, Coverage, and Analysis

**Figure 3:**
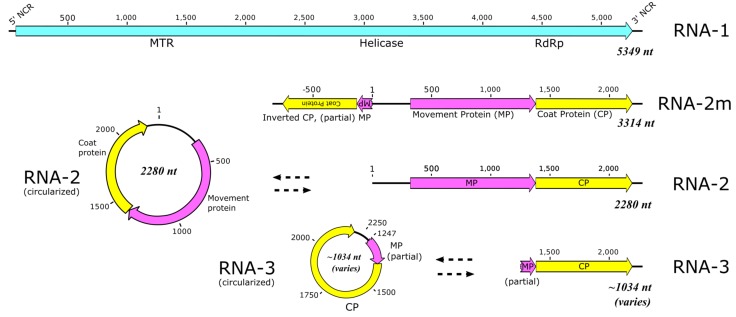
Schematic diagrams showing the proposed genome components of blackcurrant leaf chlorosis associated virus (BCLCaV), in order of size. The dotted arrows indicate direct relationships of corresponding components but not origin, as RNA-2m may be the source of the circular RNAs. The letters within the subgenomic component of RNA-2m are inverted to indicate the inverse sequence. The complete BCLCaV RNA-1 is indicated in blue; the movement protein region is indicated in purple and the coat protein (CP) region is shown in yellow.

**Figure 4:**
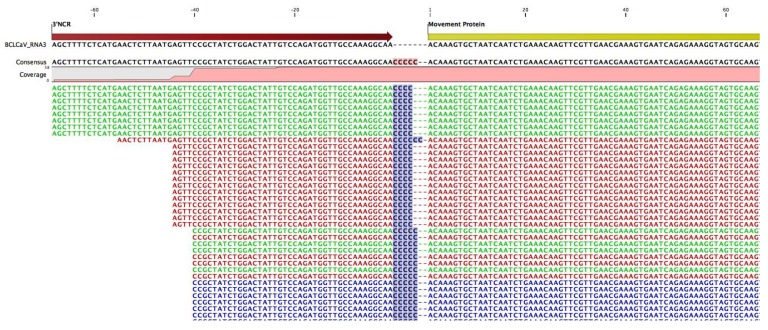
Alignment of representative and contiguous next-generation sequencing (NGS)-derived bridge reads that span the 3′ and 5′ termini of blackcurrant leaf chlorosis associated virus (BCLCaV) RNA-3. Variability in the number of cytosine (C) residues is shown at the 3′ terminus of RNA-3, resulting in the gaps observed.

Compared to RNA-1, the number of trimmed reads and the coverage associated with RNA-2 were higher by approximately 4-fold and 10-fold, respectively. In the course of analysis and genome assembly (Figure 3), bridge reads that were contiguous were identified that encompassed both the 3′-terminus and the 5′-terminus of RNA-2 or RNA-3. The proposed circular forms relative to the determined linear forms of RNA-s and RNA-3 are shown. Figure 4 shows an alignment of NGS-derived contiguous bridge reads associated with RNA-3 (5′…TTGCCAAAGGCAAC_4-7_3′/5′acaaagtgctaat …3′), with the 3′ terminus of RNA-3 in uppercase and its 5′ terminus in lowercase, showing clearly the variable number (4–7) of cytosine (C) residues at the 3′ terminus. This was observed also in RACE analysis by James and Phelan [1] (data not shown).

### 3.2. RT-PCR Verification of Bridge Reads

**Figure 5:**
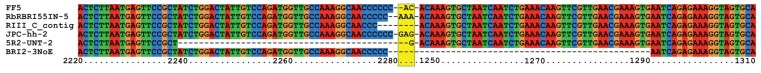
Alignment of contiguous truncated sequences of the 444 bp product generated by nested RT-PCR using primer pair BRI-4 then BRI-1 to show the region flanking the junction of the 3′ and 5′ termini of blackcurrant leaf chlorosis associated virus (BCLCaV) RNA-3. Variability in the number of cytosine (C) residues at the 3′ terminus of RNA-3 is shown and the unexpected nucleotide insertions observed are boxed. Unexpected nucleotide insertions are indicated by a yellow background. The bases A, T, G and C are indicated by backgrounds of red, green, orange and blue, respectively.

The bridging primers successfully amplified their contiguous target regions, confirming linkage. Figure 5 shows a truncated alignment of sequences of the 444 bp product that encompass the junction of the 3′ and 5′ termini of RNA-3, obtained using nested RT-PCR with the primer pairs BRI-4 and BRI-1 (Table 2, Figure 1B). Variable numbers of C residues at the 3′ terminus of RNA-3 are shown (Figure 5). Some clones contained unexpected nucleotide insertions of AC, AAA, G, or GAG, consistent with the NGS-generated reads. Interestingly, in folding analyses the associated predicted secondary structures were not affected by the insertions, including the stem loops associated with the 3′ terminus of RNA-2/RNA-3 [1,2,3]. Some clones were truncated at the 3′ terminus (Figure 5, clone 5R2-UNT-2), while other clones showed truncation at the 5′ terminus (Figure 5, clone BRI2-3NoE). Truncation at the 5′ terminus did not affect the 3′ terminus stem loops, while truncation of the 3′ terminus did not affect any remaining stem loops.

### 3.3. RT-PCR with Abutting Primers

**Figure 6:**
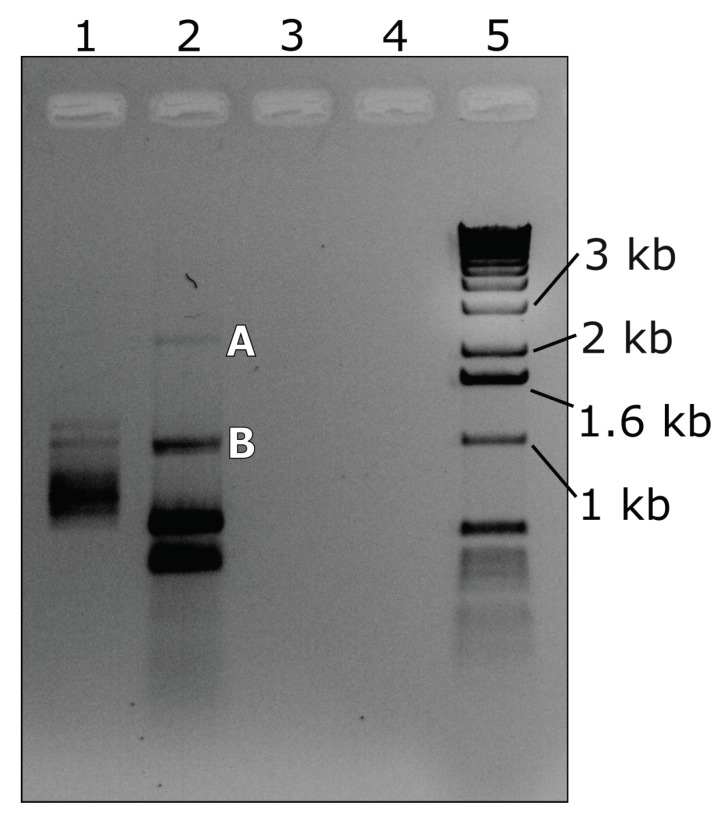
Agarose gel showing RT-PCR amplification of blackcurrant leaf chlorosis associated virus (BCLCaV) RNA-2 (lane 2, A, 2.3 kb) and RNA-3 (lane 2, B, 1.0 kb) using the abutting primer pair A. The bands A and B in lane 2 were excised, cloned, and Sanger sequenced and identified as RNA-2 (2.3 kb) and RNA-3 (1.0 kb), respectively. Lanes contain samples as follows: lane 1, dsRNA extracted from BCLCaV-infected *Ribes nigrum*; lane 2, total RNA extracted from BCLCaV-infected *R. nigrum*; lane 3, water control; lane 4, blank lane; lane 5, Invitrogen 1 kb DNA ladder.

The complete RNA-2 and RNA-3 sequences could be generated using abutting primers. Figure 6 shows the results of RT-PCR analysis of total RNA extracted from *R. nigrum* infected with BCLCaV (3124-03D1), using the abutting primer pair A (Table 3, Figure 1A). Both products A and B (Figure 6, lane 2) were cloned and Sanger sequenced and determined to be 2280 bp corresponding to RNA-2 and 1034 bp corresponding to RNA-3, respectively. Amplifications were duplicated at least twice and confirmed by cloning and Sanger sequencing. RNA-2-associated products of variable sizes were observed due to the variable 3′ terminus C residues (4–7), while in the case of RNA-3, junction site variability at both the 5′ terminus and 3′ terminus resulted in products of various sizes (937 bp, 1034 bp, and 1168 bp). Cloning and Sanger sequencing were always used to confirm size and identity.

### 3.4. RNase R Enzyme Treatments Followed by RT-PCR Analysis

**Figure 7:**
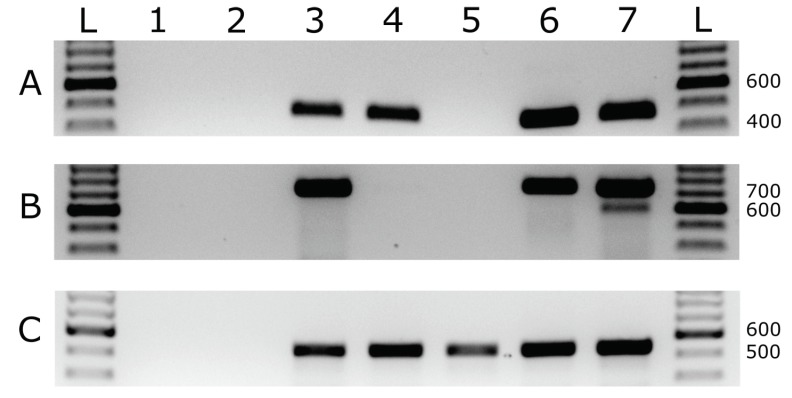
Agarose gel analysis of RT-PCR results after RNase R digestions (at 37 °C for 45 min) of dsRNA extracts from *Ribes nigrum* co-infected with blackcurrant leaf chlorosis associated virus (BCLCaV) and Actinidia virus X (AVX). Gel slice (**A**) shows RT-PCR testing for AVX using primers RivCP2F/RivXCP4R; gel slice (**B**) shows RT-PCR testing for BCLCaV RNA-1 using primers RNA1 3RACE1 and Var0; and gel slice (**C**) shows the RT-PCR testing for BCLCaV RNA-2/RNA-3 using primers RNA2 3RACE1 and CP1R. Primer sequences are provided in Table 1. Lanes L contain Invitrogen TrackIt 100 bp DNA Ladder; lane 1, uninfected *R. nigrum* negative control; lane 2, no template water control; lane 3, RT-PCR after one round of RNase digestion, lane 4, RT-PCR after two rounds of RNase R digestion; lane 5, RT-PCR after three rounds of RNase R digestion; lane 6, RT-PCR after three rounds of mock digestion (no RNase R added); lane 7, untreated positive control not subjected to mock digestions.

RNase R treatments were carried out on total RNA and dsRNA extracts from *R. nigrum* and from *N. benthamiana* co-infected with AVX and BCLCaV, and also from healthy *R. nigrum* and *N. benthamiana* used as negative controls. Results were similar and consistent. Table 4 provides a summary of tests conducted. NAD5 was not detectable after 2 rounds of RNAse R digest (Table 4). Figure 7 shows results obtained with the treatment of dsRNA extracts from healthy *R. nigrum* and from *R. nigrum* co-infected with BCLCaV and AVX. AVX was not detectable after three rounds of digestion (Table 4, Figure 7A), BCLCaV RNA-1 was not detectable after two rounds of digestion (Table 4, Figure 7B), while BCLCaV RNA-2/RNA-3 were detectable even after three rounds of digestion (Table 4, Figure 7C). All controls and control treatments gave the expected results (Table 4, Figure 7). Expected product sizes are given in Table 1.

Sequencing of post-digest abutted RT-PCR results revealed various BCLCaV sequences smaller than the full RNA2 or RNA3, which nonetheless contained a “bridge site” suggestive of a contiguously circular molecule. Nondigested samples subjected to RT-PCR also produced sequences consistent with these atypically sized circular molecules in limited instances, but the ~1034 nt sequences resulting from a “predominant bridge area” centered around nt 1247 of RNA2 were seen in most cases.

## 4. Discussion

RNA-2/RNA-3 contiguous bridging (3′ terminus/5′ terminus) paired-end reads were identified among NGS sequences generated from dsRNA extracted from *R. nigrum* cv. Baldwin (accession 3124-03D1) infected with BCLCaV. James and Phelan [1] described a concatenated form of BCLCaV genome consisting of RNA-2 linked to a complementary and inverse RNA-3, with the consequence of the 3′-NCR at both termini, similar to the RNA-2m described by Quito-Avila et al. [12] for an Ecuadorean isolate of RBDV. The BCLCaV RNA-2m bridge read consists of nts TAG CAC TTT/ATA TAT TTT, where TAG CAC TTT represents the complementary and inverse sequence of the RNA-2 ORF1 nt sequence at position 1249–1257, within the putative MP coding region. The 5′ terminus of RNA-2 is indicated in bold. The 3′ terminus of RNA-2/RNA-3 (CAA AGG CAA CCC CCC C) is not represented in this linkage.

The bridge reads described in this study are not associated with the bridge of BCLCaV RNA-2m (inverted RNA-3 conjoined to RNA-2). In this study, the bridge reads are contiguous and contain the 3′ terminus of RNA2/RNA-3 (including the variable poly(C) tail) and the 5′ terminus of the RNA-2 or of the RNA-3. This indicates a linkage that may be the result of end-to-end concatenation of BCLCaV RNA-2 and/or RNA-3 elements or associated with circular RNA. Very sensitive touchdown RT-PCRs with primers that span linkage sites were used to determine if indeed end-to-end concatenation existed, which should manifest by cDNA products or more than one cDNA products that could be amplified and sequenced to confirm concatenation. Evidence for end-to-end concatenation was never obtained. Circular RNAs are uniquely characterized by covalent linking of the 5′ and 3′ ends [14]. RT-PCRs with primers that flank the junction site did confirm the contiguous nature of the 3′ terminus/5′ terminus of BCLCaV RNA-2/RNA-3, observed in NGS paired-end reads. Interestingly, in some cases, unexpected nucleotide insertions were observed at the 3′/5′ junction site of some NGS bridge reads. They were observed also in some cases when the products of bridge RT-PCRs were cloned and sequenced, confirming their presence. These insertions had no effect on the predicted secondary structures, but the origins of these inserted nucleotides are unclear at this time.

Evidence was obtained that is consistent with the existence of circular RNA associated with BCLCaV RNA-2/RNA-3. In addition to confirmation of the bridge reads, outward facing or abutting primers were used to generate full-length products of both RNA-2 and RNA-3. Abutting primers will only generate the full-length sequence of a target if that target is circular [15,24,25,40]. Rigorous RNase R treatments did not eliminate the RNA-2/RNA-3 template. RNase R is a 3′ to 5′ exoribonuclease that has the capacity to destroy linear and branched or structured RNA, leaving only circular RNA [27,41]. In this study, dsRNA and total RNA samples from plants co-infected with BCLCaV and AVX were subjected to three rounds of denaturation and RNase R digests. BCLCaV RNA-1 [1], AVX [30,31], and the host endogenous NADH dehydrogenase subunit 5 mRNA [38] are not known to possess any circular RNA forms and could not be detected by RT-PCR after the RNAse R treatments. Only BCLCaV RNA-2/RNA-3 could be detected after three consecutive rounds of RNase R treatments. Single-stranded RNA (ssRNA) viruses usually have a replicative stage in the form of double-stranded RNA (dsRNA) [42]. Denaturation at 95 °C for 8 min was used to ensure that dsRNAs were converted to linear ssRNA targets.

The multiple forms of BCLCaV RNA-2/RNA-3 is likely a contributing factor to the much higher NGS read coverage associated with RNA-2/RNA-3, compared to the RNA-1-associated reads. Interestingly, this may facilitate enhanced reliability of detection using tests such as RT-PCR that target the CP encoding region present in these various RNA-2/RNA-3 forms. This is important, as BCLCaV is associated with disease symptoms [1].

Base-pairing of flanking and complementary sequences can serve to facilitate circularization [16,30]. BCLCaV RNA-2m, which consists of the RNA-2 and an inverted and complementary RNA-3, has the potential to form a loop or hairpin similar to that proposed for the RNA-2m of RBDV [12]. It is quite possible, therefore, that BCLCaV RNA-2m plays a role in the genesis of the circular RNA observed. Herold and Andino [13] indicated that RNA circularization may play a role in the replication of positive sense RNA viruses. The role that circular RNA may play in the replication and/or translation processes of BCLCaV is unknown at this time. Circular RNA is described as having several properties and/or functions including: RNA stabilization and protection from degradation by ubiquitous RNases [43]; acting as a microRNA sponge, involvement in protein binding, regulation of translation and translation into proteins [18]; serving as templates for rolling circle replication, a highly efficient way of generating many copies of a specific RNA [23]. There are also small (220–288 nt), circular satellite RNAs that are known to be associated with plant-infecting ssRNA viruses [44]. Interestingly, RT-PCR with abutting primers post-RNase R digestion revealed some smaller contiguous BCLCaV-derived RNA-2 sequences but further analysis is required.

If there is indeed any accuracy to the statement by Herold and Andino [13] that circularization may play a role in the replication of positive sense RNA viruses, then with the increasing use of NGS as an analytical and diagnostic tool [45,46,47], it is quite likely that further discoveries of circular RNA will occur. RNA-2m has been described for an Ecuadorean isolate of RBDV [12] and for BCLCaV [1]. It would be interesting to know if this RNA-2m form is associated with other isolates of RBDV and with PrLBaV, the other proposed new member of the genus *Idaeovirus* [3]. Further studies, such as 2D gel electrophoresis [18], will be carried out to further confirm circularization.

Pettit Kneller et al. [48] stated that two features are necessary for efficient translation of mRNAs—a 5′ m^7^G(5′)ppp(5′) N cap and a 3′ poly(A) tail—and that, in some cases, as in the case of potyviruses, a 5′ VPg might act in a manner similar to the 5′ cap. RBDV [4] and the two proposed new members of the genus *Idaeovirus*, PrLBaV [3] and BCLCaV [1,2], lack any 5′ cap, any 5′ VPg, or any 3′ poly(A) tail. They possess 3′ poly(C) tails, with conserved hexanucleotides (AUAUCU) at the 5′ termini [1,2,3]. It seems that the first and third position of the hexanucleotide (AxAxxx) may be the critical positions, as only these are conserved in BCLCaV RNAs [1]. Although BCLCaV-associated putative circular RNA-2 and RNA-3 contain ORFs with stop and start codons in frames, it is unknown if they are translated. Recently, Pamudurti et al. [49] provided definitive evidence of both in vitro and in vivo cap-independent translation of some circular RNA. Perhaps RNA circularization may play a role in an alternate cap-independent translation mechanism.

Holmes [50] indicated that tools such as NGS will increase our knowledge of the virosphere. New and interesting virus species are likely to be discovered in new and unexpected places, with new and interesting relationships. NGS facilitated the identification of BCLCaV RNA-2/RNA-3 bridge reads. It is possible also that a greater range of virus diversity and genome organizations will be discovered and determined to be more common than first appears. New insights may be gained into our understanding of virus relationships, their organization, and their evolution.

## Figures and Tables

**Table 1:** Diagnostic primers used to screen for various targets, including blackcurrant leaf chlorosis associated virus (BCLCaV) RNA-1 and RNA-2/RNA-3.

Primer Pair	Primer Names		5′–3′ Sequence	Location of Primer Binding Sites	Expected RT-PCR Product Size (nt)
NAD5 ^1^	NAD5-F	(F)	GATGCTTCTTGGGGCTTCTTGTT	Host mRNA; NADH dehydrogenase-5 gene	181
	NAD5-R	(R)	CTCCAGTCACCAACATTGGCATAA		
AVX ^2^	RivCP2F	(F)	CAGCTTGTACGAGCGTATG	Actinidia virus X; coat protein region	460
	RivXCP4R	(R)	AGCTAGGTTGGAGATGTAATTG		
Diagnostic RNA-1 ^3^	RNA1 3RACE1	(F)	GAGCCAAGCTCACAAACACTC	4625 to 4645 (RNA1)	710
	Var0	(R)	GGCAACCATCTGGACAATAG	5315 to 5334 (RNA1)	
Diagnostic RNA-2 ^2^	RNA2 3RACE1	(F)	ACCAGCATTTCGCAGTTCAG	1599 to 1618 (RNA2)	528
	CP1R	(R)	ATTCTACCCAGCGCCGTAAG	2107 to 2126 (RNA2)	

^1^ NAD5 (mRNA transcribed from host NAD5 endogenous gene) primers described by Menzel et al. [38]. ^2^ AVX (Actinidia virus X) primers described by James and Phelan [31] and ^3^ BCLCaV primers described by James and Phelan [1].

**Table 2:** Oligonucleotide primers used to confirm bridging of the 3′ terminus and 5′ terminus of blackcurrant leaf chlorosis associated virus (BCLCaV) RNA-2/RNA-3.

Primer Pair	Primer Names		5′–3′ Sequence	Location along BCLCaV RNA2	Expected RT-PCR Product Size (nt) ^1^
BRI-0	RNA2 3RACE3	(F)	TGTTGCGGTGGTTGAAGTTG	1909 to 1928	722 (full RNA-2 target)
	RNA2 5RACE3	(R)	CGAACGCATACTCACTGAAC	331 to 350	
BRI-1	RNA2 3RACE3	(F)	TGTTGCGGTGGTTGAAGTTG	1909 to 1928	444 (RNA-3 target)
	Var4	(R)	GGCTGACTTGCACTACCTTTC	1298 to 1318	
BRI-4	RNA2 3RACE2	(F)	TGCCTGAGGGAGAGGTTGTG	1701 to 1720	689 (RNA-3 target)
	1355R	(R)	GGCTTTCTGCGGTATTGGTC	1336 to 1355	

^1^ RT-PCR products sometimes varied from the expected size due to variation in the number of C residues (4–7) at the 3′ terminus and variability at the 5′ terminus.

**Table 3:** Abutting primers used for analysis and screening of blackcurrant leaf chlorosis associated virus (BCLCaV) RNA-2/RNA-3 targets.

Primer Pair	Primer Names		5′–3′ Sequence	Location along BCLCaV RNA2	Expected (RNA-3) RT-PCR Product Size ^1^ (nt)
Abutting pair A	RNA2 3RACE2	(F)	TGCCTGAGGGAGAGGTTGTG	1701 to 1720	1034
	Var2	(R)	AACCCGAGTGGTAGAGGAG	1681 to 1699	
Abutting pair B	1356F	(F)	CCGAACCCTATTACTAGGATCTG	1356 to 1378	1034
	1355R	(R)	GGCTTTCTGCGGTATTGGTC	1336 to 1355	
Abutting pair C	1519F	(F)	ACCCTTCGCTAGTGTGGATCTC	1519 to 1540	1034
	Var3 ^2^	(R)	TCACCCGTCGCTGGATTAG	1500 to 1518	

^1^ RT-PCR products sometimes varied from the expected size due to variation in the number of C residues (4–7) at the 3′ terminus and variability at the 5′ terminus. ^2^ Primers described by James and Phelan [1].

**Table 4:** Summary of RNase R digestion studies with RT-PCR tests performed after each of three rounds of digestion to determine presence of the target.

RT-PCR Test	Topology of Target	Strandedness of Target	Post Digest PCR Amplification			Amplification of All No-Enzyme Controls
			After 1 Round	After 2 Rounds	After 3 Rounds	
NAD5 ^1^	Linear	ssRNA	not done	N ^4^	not done	Y ^5^
AVX ^2^	Linear	ssRNA with dsRNA phase	Y	Y	N	Y
BCLCaV ^3^ RNA1 diagnostic	Linear, not known to be circular	ssRNA with dsRNA phase	Y	N	N	Y
BCLCaV RNA2 diagnostic	linear + hypothesized circular	ssRNA with dsRNA phase	Y	Y	Y	Y

^1^ NAD5 = mRNA transcribed from host NAD5 endogenous gene; ^2^ AVX = Actinidia virus X; ^3^ BCLCaV = blackcurrant leaf chlorosis associated virus, ^4^ N = no amplification observed, ^5^ Y = amplification observed.
